# Personalized pancreatic cancer therapy: from the perspective of mRNA vaccine

**DOI:** 10.1186/s40779-022-00416-w

**Published:** 2022-10-13

**Authors:** Xing Huang, Gang Zhang, Tian-Yu Tang, Xiang Gao, Ting-Bo Liang

**Affiliations:** 1 Zhejiang Provincial Key Laboratory of Pancreatic Disease, the First Affiliated Hospital, Zhejiang University School of Medicine, Hangzhou, 310009 China; 2 Department of Hepatobiliary and Pancreatic Surgery, the First Affiliated Hospital, Zhejiang University School of Medicine, Hangzhou, 310003 China; 3 Zhejiang Clinical Research Center of Hepatobiliary and Pancreatic Diseases, Hangzhou, 310003 China; 4 The Innovation Center for the Study of Pancreatic Diseases of Zhejiang Province, Hangzhou, 310009 China; 5 Cancer Center, Zhejiang University, Hangzhou, 310058 China

**Keywords:** Pancreatic cancer, Precise therapy, Cancer vaccine, mRNA vaccine, Tumor antigen, Immune subtype

## Abstract

Pancreatic cancer is characterized by inter-tumoral and intra-tumoral heterogeneity, especially in genetic alteration and microenvironment. Conventional therapeutic strategies for pancreatic cancer usually suffer resistance, highlighting the necessity for personalized precise treatment. Cancer vaccines have become promising alternatives for pancreatic cancer treatment because of their multifaceted advantages including multiple targeting, minimal nonspecific effects, broad therapeutic window, low toxicity, and induction of persistent immunological memory. Multiple conventional vaccines based on the cells, microorganisms, exosomes, proteins, peptides, or DNA against pancreatic cancer have been developed; however, their overall efficacy remains unsatisfactory. Compared with these vaccine modalities, messager RNA (mRNA)-based vaccines offer technical and conceptional advances in personalized precise treatment, and thus represent a potentially cutting-edge option in novel therapeutic approaches for pancreatic cancer. This review summarizes the current progress on pancreatic cancer vaccines, highlights the superiority of mRNA vaccines over other conventional vaccines, and proposes the viable tactic for designing and applying personalized mRNA vaccines for the precise treatment of pancreatic cancer.

## Background

The annual pancreatic cancer cases have doubled over the past two decades, increasing from 196,000 patients worldwide in 1990 to 441,000 in 2017 [1]. According to the 2020 global cancer statistics, there were 495,773 new cases of pancreatic cancer [2]. Given the increase in life expectancy of the global population, the incidence of pancreatic cancer is expected to continue rising over the coming decades. Surgical intervention is currently the only curative option for pancreatic cancer management in the clinic. However, only 15–20% of patients qualify for the corresponding surgery, attributed to the limited routine screening methods for detecting pancreatic cancer at an early stage [3]. Moreover, despite complete resection, local or distant recurrence of pancreatic cancer is often observed within two years after surgery [4]. Systematic chemotherapy has been the standard treatment for more than 80% of patients with locally advanced diseases or distant metastases for several decades. Even though gemcitabine plus nab-paclitaxel and FOLFIRINOX are the most recommended chemotherapeutic regimens for metastatic pancreatic ductal adenocarcinoma (PDAC) treatment, acquired resistance against these drugs is common [5–7]. Immunotherapy, targeted therapy, and other promising treatments have also been tested in preclinical studies and clinical trials; however, almost all strategies show little significant advantage over conventional chemotherapy against pancreatic cancer, together with the prevalent therapeutic resistance [8, 9]. Accordingly, the overall 5-year survival of pancreatic cancer patients is only about 10%, making the tumor is one of the leading causes of cancer-related mortality [10]. Obviously, there is an urgent need for highly effective alternatives for pancreatic cancer treatment.

Accumulating evidence indicates that the therapeutic resistance in pancreatic cancer is associated with its inter-tumoral and intra-tumoral heterogeneity, particularly as regards the genetic alteration and immune microenvironment [11–13]. For instance, *SMAD4* mutation occurs in about 50% of PDAC patients [14], and this mutation promotes radiotherapeutic resistance by increasing the production of reactive oxygen species and inducing autophagy [15]. Appropriately 6% of pancreatic cancers display *BRCA1/2* or *PALB2* mutations [16, 17], and the lack of mutations in these genes is associated with resistance to platinum-based chemotherapy [17, 18]. Tumors without *BRCA1/2* mutations are also susceptible to generating PARP inhibitor resistance [19–21]. In addition, the inter-tumoral heterogeneity in the immune microenvironment promotes resistance to immunotherapy [11]. Taking the programmed cell death 1/programmed cell death ligand 1 (PD-1/PD-L1) blockade as an example, its therapeutic efficacy is associated with the pre-infiltration of T cells [22]. Only < 1% of PDAC patients with high microsatellite instability that was detected the presence of neoantigen-specific T cell immunity in tumor respond to PD-1 inhibition [23–26]. In contrast, most tumors are characterized by low immunogenicity and lack of T cell infiltration and are thus resistant to immunotherapy targeting PD-1/PD-L1. Notably, pancreatic cancer is classified into distinct subtypes based on gene expression or immune characteristics [27–29]. For instance, Moffitt et al. [28] identified two stromal subtypes (normal and activated) and two tumor subtypes (basal-like and classical) based on gene expression profiles. Compared with classical subtype tumors, basal-like subtype tumors exhibit a superior response to adjuvant chemotherapy. Apart from inter-tumoral heterogeneity, increasing evidence has uncovered the intra-tumoral heterogeneity in pancreatic cancer [30]. At least three types of intra-tumoral genetic heterogeneity have been proposed [13]. Type-1 includes mutations distinguishing tumor cells within the same primary lesions, type-2 includes mutations distinguishing tumor cells within the same metastatic lesions, while type-3 includes mutations distinguishing tumor cells among different metastatic lesions. The intra-tumoral heterogeneity largely promotes adaptive resistance to cancer therapy. Single-cell sequencing for pancreatic cancer has revealed the existence of both basal-like and classical subtypes in the same tumor, partially explaining the adaptive resistance to chemotherapy [31]. These reports highlight the heterogeneity-induced therapeutic resistance, underlining the significance of developing personalized precise treatment against pancreatic cancer. Compared with the traditional monoclonal antibodies and small molecule inhibitors, cancer vaccines offer several advantages, including minimal nonspecific effects, broad therapeutic window, low toxicity, and induction of persistent immunological memory [32, 33]. Moreover, cancer vaccines can achieve precise targeting based on the characteristics in individual tumors. Therefore, vaccination is a potential approach for personalized pancreatic cancer treatment, overcoming the challenges posed by tumor heterogeneity.

Messager RNA (mRNA) vaccine has recently become one of the most potent vaccine types in prevention and treatment of multiple diseases. The successful development of mRNA vaccine is attributed to decades of relentless and intensive research. mRNA was discovered in 1961 and isolated for in vitro protein expression in 1969 [34, 35]. Until 1990, in vitro transcribed mRNA was validated able to be template to produce proteins in mouse skeletal muscle cells in vivo [36]. This was the first successful attempt for in vivo mRNA expression, setting the stage for mRNA vaccine development. Later in 1992, mRNA for vasopressin was injected and expressed in the hypothalamus, inducing physiological responses [37]. Thereafter in 1993 and 1995, mRNA was reported to induce both cellular and humoral immunity [38–40]. However, these promising findings did not attract substantial investment in the development of mRNA vaccines largely due to the perceived mRNA instability, inefficient in vivo delivery, and potential innate immunogenicity. Given the safety, simple design, and ease of manufacturing, research on mRNA continued. The technological advances in the modification and delivery of mRNA largely addressed these concerns. For instance, the application of modified nucleosides prevents mRNA recognition by pattern recognition receptors (PRRs), enhancing the translational efficacy [41]. Application of vehicles (e.g., lipid nanoparticle, polyplexes, and polymeric nanoparticles) promotes the in vivo delivery of mRNA [42]. The improvement of in vivo translational efficiency and delivery enhances the chance of clinical application of mRNA vaccines. The first application of personalized mRNA vaccine in humans was reported in 2017 against melanoma, and the vaccination induced specific immune activation, decreased the metastatic rate and prolonged the progression-free survival of patients [43]. In addition, multiple clinical trials on the efficacy of mRNA-based vaccination against human immunodeficiency virus have been completed [44]. Since its outbreak in 2019, severe acute respiratory syndrome coronavirus-2 (SARS-CoV-2), the cause of the coronavirus disease 2019 (COVID-19), has infected and caused millions of deaths globally [45, 46]. Due to the threat posed by SARS-CoV-2, various treatments were rapidly developed to contain the spread of the virus. Owing to the convenience in mass production and advances in modification techniques of mRNA, two mRNA vaccines, BNT162b2 and mRNA-1273, obtained the authorization of emergency use for preventing COVID-19 [47–50]. Both achieved a protective efficacy of over 90%, and were officially approved for mass vaccination by the Food and Drug Administration against SARS-CoV-2 [49–53]. Of note, SARS-CoV-2 underwent multiple mutations, compromising the protective efficacy of BNT12b2 and mRNA-1273 [54–56]. However, this concern has currently been solved to a large extent by updating vaccine-encoded antigens accordingly, together with optimizing the administration of vaccination and boosting. The strategies used for overcoming SARS-CoV-2 variations provide valuable experience for developing and applying the personalized anti-pancreatic cancer mRNA vaccine. Together, the application of mRNA vaccine in other diseases lays a foundation for the development of personalized anti-pancreatic cancer mRNA vaccine.

This review summarizes the current advances and status of pancreatic cancer vaccines, emphasizes the superiority of mRNA-based vaccines in cancer precision treatment, and highlights the strategy for developing personalized mRNA vaccines against pancreatic cancer.

## Conventional pancreatic cancer vaccines

**Fig. 1:**
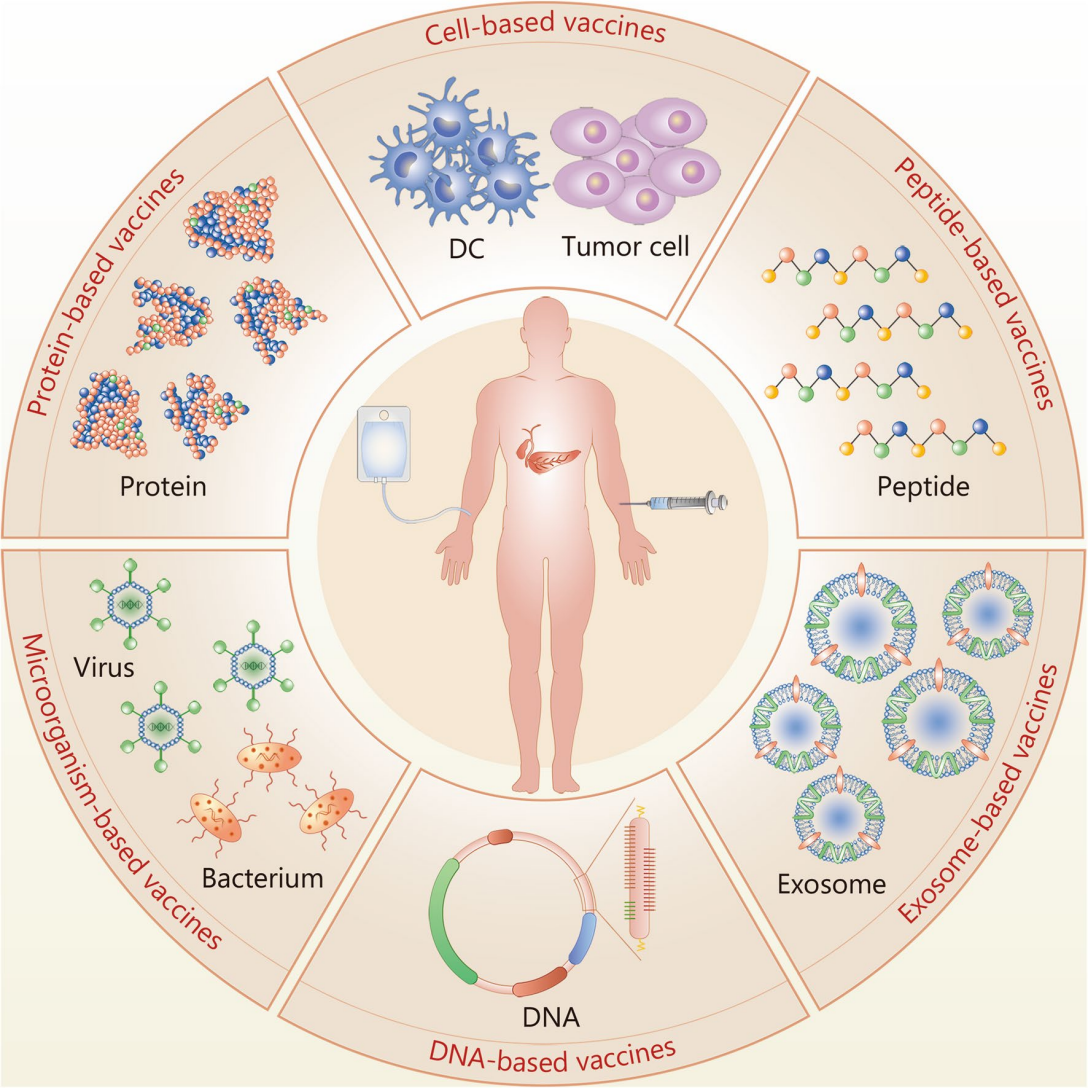
Classification of existing pancreatic cancer vaccines. Multiple pancreatic cancer vaccines have been developed to date, including cell-based vaccines, microorganism-based vaccines, exosome-based vaccines, protein-based vaccines, peptide-based vaccines, and DNA-based vaccines. DC dendritic cell

To date, multiple conventional vaccines against pancreatic cancer, including cell-based, microorganism-based, exosome-based, protein-based, peptide-based, and DNA-based forms (Fig. 1), are under development (completed clinical trials are summarized in Table 1, and ongoing clinical trials in Table 2).

**Table 1:** Completed clinical trials of pancreatic cancer vaccines

Vaccine type	NCT number	Immunogen	Additional treatment	Phase	Enrollment	Endpoint
Cell-based vaccine	NCT00004604	CEA RNA-pulsed autologous DC	No	Phase I	Not provided	2002
	NCT00002773	Allogeneic pancreatic cancer cell	Cyclophosphamide, GM-CSF	Phase II	Not provided	2004
	NCT00084383	GVAX	5-fluorouracil, radiotherapy	Phase II	60	2006
	NCT00255827	Allogeneic tumor cell expressing α-1,3 galactosyltransferase	No	Phase I/II	7	2007
	NCT00128622	Autologous DC-infected with fowlpox-CEA-6D-TRICOM	Denileukin diftitox	Phase I	Not provided	2007
	NCT00027534	Autologous DC-infected with fowlpox-CEA-6D-TRICOM	Autologous DC mixed with CMV pp65 and tetanus toxoid	Phase I	Not provided	2007
	NCT00547144	Autologous DC	Gemcitabine, stereotactic radiosurgery	Phase I	2	2008
	NCT00002475	Allogeneic or autologous tumor cell	Cyclophosphamide, GM-CSF	Phase II	Not provided	2009
	NCT00305760	GVAX	Cetuximab, cyclophosphamide	Phase II	60	2009
	NCT00161187	Allogeneic lymphocyte	No	Phase I	Not provided	2011
	NCT01410968	Peptide-pulsed DC	Poly-ICLC	Phase I	12	2016
	NCT02151448	Autologous αDC1-loaded with autologous tumor material	Celecoxib, IFN-α, rintatolimod	Phase I/II	64	2019
	NCT00727441	GVAX	Surgery, cyclophosphamide	Phase II	87	2019
	NCT01896869	GVAX	FOLFIRINOX, ipilimumab	Phase II	83	2019
Peptide-based vaccine	NCT00006387	RAS	Immunological adjuvant QS21	Phase I	Not provided	2002
	NCT00008099	MUC1	SB AS-2	Phase I	25	2004
	NCT00019006	RAS	Detox-B adjuvant	Phase I	Not provided	Not provided
	NCT00019331	RAS	IL-2, GM-CSF	Phase II	Not provided	2007
	NCT00648102	HCG-β	No	Phase I	Not provided	2009
	NCT00622622	VEGFR2	Gemcitabine	Phase I	21	2009
	NCT00709462	HCG-β	No	Phase I	Not provided	2010
	NCT00529984	CEA	No	Phase I/II	Not provided	2010
	NCT00425360	Telomerase	Gemcitabine, capecitabine, GM-CSF	Phase III	Estimated 1110	2013
	NCT00655785	VEGFR1, VEGFR2	Gemcitabine	Phase I/II	17	2013
	NCT01342224	Telomerase	GM-CSF, gemcitabine	Phase I	11	2018
Microorganism-based vaccine	NCT00003125	ALVAC-CEA, vaccinia-CEA	IL-2, GM-CSF	Phase II	Not provided	2004
	NCT00028496	Fowlpox-CEA(6D)-TRICOM	GM-CSF	Phase I	Not provided	2005
	NCT01191684	MVAp53	No	Phase I	Not provided	2013
	NCT00569387	Algenpantucel-L	Surgery, gemcitabine and 5-fluorouracil	Phase II	73	2014
	NCT00300950	Yeast expressing four different mutated RAS protein	Gemcitabine	Phase II	176	2015
	NCT02338752	DPT, typhoid, staphylococcus aureus, paratyphoid A and B	Surgery, chemotherapy	Phase I/II	20	2015
	NCT03127098	Adenovirus [E1-, E2b-]-CEA(6D)	IL-15	Phase I/II	Not provided	2017
Protein-based vaccine	NCT00003025	HSPPC-96	No	Phase I	16	2002
DNA-based vaccine	NCT01486329	VEGFR-2 DNA	No	Phase I	72	2014

All clinical trial data were collected from ClinicalTrials.gov (https://clinicaltrials.gov/ct2/home). *CEA* carcinoembryonic antigen, *DC* dendritic cell, *GM-CSF* granulocyte–macrophage colony-stimulating factor, *GVAX* GM-CSF gene-transfected allogeneic pancreatic cancer cell, *CMV pp65* cytomegalovirus pp65, *αDC1* α-type-1 polarized dendritic cell, *RAS* Ras GTPase-activating protein, *MUC1* mucin 1, *VGEFR* vascular endothelial growth factor receptor, *HCG-β* human chorionic gonadotropin beta, *SB AS-2* an immunologic adjuvant system consisting of an oil-in-water emulsion containing two immunostimulants: monophosphoryl Lipid A and a saponin derivative QS-21, *MVAp53* modified vaccinia virus ankara vaccine expressing p53, *DPT* diphtheria, pertussis, tetanus, *HSPPC-96* heat shock protein-peptide complex-96

**Table 2:** Ongoing clinical trials of pancreatic cancer vaccines

Vaccine type	NCT number	Immunogen	Additional treatment	Phase	Estimated enrollment	Status	Start point
Cell-based vaccines	NCT00389610	GVAX	No	Phase II	56	Active, not recruiting	2006
	NCT01088789	GVAX	Cyclophosphamide	Phase II	72	Recruiting	2010
	NCT01595321	GVAX	SBRT, FOLFIRINOX, cyclophosphamide	Not applicable	19	Active, not recruiting	2012
	NCT02451982	GVAX	Cyclophosphamide	Phase II	76	Recruiting	2016
	NCT02648282	GVAX	Cyclophosphamide, pembrolizumab, SBRT	Phase II	58	Active, not recruiting	2016
	NCT03190265	GVAX	Cyclophosphamide, nivolumab, CRS-207, ipilimumab	Phase II	63	Active, not recruiting	2017
	NCT03161379	GVAX	SBRT, nivolumab, cyclophosphamide	Phase II	30	Active, not recruiting	2018
	NCT03592888	Autologous DC pulsed with mutant KRAS peptides	No	Phase I	12	Recruiting	2018
	NCT03006302	GVAX	Epacadostat, pembrolizumab, CRS-207, cyclophosphamide	Phase II	40	Active, not recruiting	2018
	NCT03153410	GVAX	Cyclophosphamide, pembrolizuma, IMC-CS4	Phase I	12	Active, not recruiting	2018
	NCT03767582	GVAX	SBRT, nivolumab, CCR2/CCR5 dual antagonist	Phase I/II	30	Recruiting	2019
	NCT04157127	Autologous DC loaded with tumor lysate plus mRNA	No	Phase I	43	Recruiting	2020
	NCT04627246	Autologous DC loaded with personalized peptides	Nivolumab, chemotherapy	Phase I	12	Recruiting	2020
Peptide-based vaccines	NCT03558945	Personalized neoantigen	Poly-ICLC	Phase I	60	Recruiting	2018
	NCT04161755	Personalized neoantigen	Atezolizumab, surgery, FOLFIRINOX	Phase I	29	Active, not recruiting	2019
	NCT04117087	KRAS	Nivolumab, ipilimumab	Phase I	30	Recruiting	2020
	NCT03956056	Personalized neoantigen and mesothelin	Poly-ICLC	Phase I	12	Active, not recruiting	2020
	NCT04810910	Personalized neoantigen	Surgery, chemotherapy	Phase I	20	Recruiting	2021
	NCT05111353	Neoantigen synthetic long peptide	Poly-ICLC	Phase I	30	Not yet recruiting	2022
	NCT05013216	KRAS	Poly-ICLC	Phase I	25	Recruiting	2022
Microorganism-based vaccines	NCT00669734	Vaccinia, fowlpox	GM-CSF	Phase I	18	Active, not recruiting	2010
	NCT03136406	Recombinant saccharomyces cerevisiae yeast expressing mutant Ras	Cyclophosphamide, oxaliplatin, GI-4000, capecitabine, 5-fluorouracil, leucovorin, nab-paclitaxel, aNK, bevacizumab, avelumab, ALT-803, ETBX-011	Phase I/II	3	Active, not recruiting	2017
	NCT05116917	Influenza virus	Nivolumab, ipilimumab, SBRT	Phase II	30	Recruiting	2021
DNA-based vaccines	NCT03122106	Personalized neoantigens and mesothelin DNA	No	Phase I	15	Active, not recruiting	2018

All clinical trial data were collected from ClinicalTrials.gov (https://clinicaltrials.gov/ct2/home). *GM-CSF* granulocyte–macrophage colony-stimulating factor, *GVAX* GM-CSF gene-transfected allogeneic pancreatic cancer cell, *SBRT* stereotactic body radiation therapy, *CRS-207* listeria monocytogenes-expressing mesothelin, *DC* dendritic cell, *KRAS* GTPase KRas, *CCR* C–C chemokine receptor, *aNK* NK-92 cells

### Cell-based pancreatic cancer vaccines

The currently available cell-based pancreatic cancer vaccines include dendritic cell (DC)-based and tumor cell-based forms. As the most potent antigen-presenting cell (APC), DCs are usually loaded with an antigen and re-infused into patients [57]. In a phase I/II clinical trial, 12 patients with resected pancreatic and biliary cancer received mucin 1 (MUC1) peptide-loaded DC vaccine [57, 58]. Four of them survived more than 4 years after the vaccination and showed no signs of recurrence. In a related study, Wilms tumor (WT) 1-specific cytotoxic T cells were observed in seven out of eight cancer patients who received a combination of WT1-peptide-pulsed DC-based vaccine and S-1 or S-1 plus gemcitabine after surgery [59]. Of note, the procedure for developing DC vaccines is highly laborious and time-consuming and requires autologous cell preparations that do not meet economic requirement of precision therapy. Cell-based vaccines also include autologous and allogeneic tumor cell-derived vaccines [57, 59]. Even though autologous tumor cell-based vaccines are particularly suitable for personalized therapy, autologous tumor cells may be insufficient, as only 15–20% of pancreatic cancer patients are eligible for surgery [3, 57]. Therefore, allogeneic tumor cell-based vaccines, including GVAX [allogeneic granulocyte–macrophage colony-stimulating factor (GM-CSF)-secreting pancreatic cancer vaccine] and Algenpantucel-L (hyperacute-pancreatic cancer vaccine), are alternatives for pancreatic cancer treatment [57, 60, 61]. However, this approach does not consider the extensive heterogeneity of pancreatic cancer and thus is not suitable for personalized therapy. Compared with cyclophosphamide, a phase II trial revealed that GVAX did not improve the survival of patients with metastatic PDAC [60]. In one multi-institutional phase II clinical trial, the 12-month overall survival and disease-free survival rate of pancreatic cancer patients after treatment with Algenpantucel-L combined with standard adjuvant chemoradiotherapy reached 86% and 62%, respectively [60, 62].

### Microorganism-based pancreatic cancer vaccines

Microorganism-based pancreatic cancer vaccines are classified into bacteria, viruses, and recombinant yeast-based forms [63, 64]. These vaccines represent a co-expressing strategy of tumor antigens and costimulatory molecules. The human adenovirus 40-based mesothelin vaccine inhibited the growth and metastasis of pancreatic cancer in mice [65]. An open-label phase I study of advanced pancreatic cancer showed that recombinant prime-boost poxviruses (targeting MUC1 and carcinoembryonic antigen prolonged the overall survival of patients with anti-MUC1 and/or carcinoembryonic antigen-specific immune responses [66]. In contrast, a phase II clinical trial revealed that compared with chemotherapy, vaccination with live-attenuated *Listeria monocytogenes* expressing mesothelin had no significant overall survival benefits for metastatic PDAC patients [67]. Notably, microorganism-based vaccines require complicated engineering system and elaborate fabrication, undermining their inconvenient application for personalized treatment.

### Exosome-based pancreatic cancer vaccines

Tumor-derived exosomes (TEXs) are nanosized lipid bilayer encapsulating vesicles that shuttle bioactive information to the tumor microenvironment, promoting tumor progression [68, 69]. TEXs contain various tumor antigens and feature discrete sets of specific proteins that promote DC-binding and uptake of exosomes. In pancreatic cancer mouse models, DCs loaded with TEXs vaccine activated CD4^+^ T cells and significantly prolonged the survival of mice compared to cytotoxic drugs [70]. Notably, TEXs also include proteins and nucleic acids which have strong capability to boost the body’s immunity, and thus may cause auto-immune diseases by disrupting the immune homeostasis after vaccination, posing a challenge to safety of precision therapy [71–74].

### Protein-based pancreatic cancer vaccines

Proteins for cancer vaccination are not only immunogenic but also can carry additional antigenic peptide. Proteins vaccines based on heat shock proteins, especially heat shock protein-peptide complex-96 (HSPPC-96), are currently under several clinical trials to investigate their therapeutic potential against different cancers [75–80]. A phase I pilot study revealed that 30% (3/10) of patients with resected pancreatic cancer survived for more than 5 years after vaccination with the HSPPC-96 vaccine [75]. Notably, given that HSPPC-96 must be extracted from tumor tissues of each patient, its use largely depends on the resectability of the tumors [80].

### Peptide-based pancreatic cancer vaccines

Peptide-based vaccines are developed based on antigenic epitopes, the minimal immunogenic regions of antigens [81]. KRAS-targeting peptide was the first peptide-based vaccine to undergo clinical trials [82]. In a phase I/II study, GM-CSF combined with KRAS-targeting peptide vaccine-induced specific immune response in 25 of 43 (58%) patients, and the survival period was also significantly longer for responders than non-responders [83]. Another commonly tested peptide-based vaccine, the telomerase-targeting vaccine (GV1001), was well tolerated and improved patient survival in a phase I/II clinical trial [84]. However, two phase III clinical trials revealed that compared with mono-gemcitabine, a combination of GV1001 with gemcitabine did not significantly improve the overall survival of patients with advanced pancreatic cancer [85]. Aside from KRAS and telomerase-targeted peptides, clinical trials have revealed that the efficacy of survivin, gastrin, vascular endothelial growth factor receptor (VEGFR)-1, VEGFR-2, WT1, and kinesin family member 20A-targeted vaccines is unsatisfactory[86–90]. Notably, the tumor peptide vaccine is major histocompatibility complex (MHC)-restricted and only activates monoclonal T cells, which may reduce the strength of anti-tumor immune response and thus do not satisfy the need of efficiency underlying precision therapy [81].

### DNA-based pancreatic cancer vaccines

DNA-based vaccines serve as templates encoding antigens in transfected cells. Enolase 1 (ENO1), MUC1, survivin, and VEGFR-2-targeting DNA vaccines are examples of the DNA-based pancreatic cancer vaccines explored so far [91–95]. Preclinically, the ENO1 DNA vaccine efficiently induced the infiltration of effector T cells, antibody formation, and tumor cytotoxicity in genetically engineered mice with pancreatic cancer [92]. Moreover, combined with chemotherapy, the ENO1 DNA vaccine induced CD4^+^ T cell-meditated antitumor activity and strongly impaired cancer progression in mice [92]. MUC1-targeted DNA vaccine induced strong and specific cytotoxic T lymphocyte response and showed both therapeutic and prophylactic effects in mice [91]. The survivin DNA vaccine induced specific antitumor immunity and prolonged the survival period of mice [94]. Also, VXM01, an oral DNA vaccine targeting VEGFR-2, is under phase I trial for stage IV pancreatic cancer treatment [95]. Notably, DNA vaccines increase the risk of host genomic alteration, the coded antigens are expressed over a long-time, and the production of anti-DNA autoantibodies may limit their application [96, 97]. Obviously, the safety is a major concern for application of DNA vaccine in personalized pancreatic cancer treatment.

In summary, these conventional vaccines show a measure of progress in pancreatic cancer therapy. However, given the major concerns, including the safety and complexity in preparation, they are not the best options for vaccines-based personalized precise treatment of pancreatic cancer. Therefore, it is essential to select a novel kind of vaccines that meet the needs of individual pancreatic cancer patients.

## Superiority of mRNA vaccine for cancer precision treatment

mRNA vaccines are emerging as potent candidates for cancer precision treatment because of their unique advantages over the above-mentioned vaccine formats. In addition to overcoming tumor heterogeneity by encoding personalized protein according to the genetic expression profile of tumor, mRNA vaccine meets the requirements of precision therapy highlighting precise targeting, high efficiency, safety, and economic cost.

### Generation of natural protein products

As fore-mentioned, precise targeting is key for personalized therapy. Consistent with this point, mRNA functions as a template for protein translation, and utilizes the machinery in host cells for vaccine production. This characteristic allows for post-translational modification of the protein products, including proper folding for effective functioning [44, 98]. Also, this approach allows for the production of correctly folded and assembled multimeric proteins that cannot be generated in bioreactors; this method allows for the produced transmembrane and intracellular proteins to be translocated to the appropriate specific cellular sites. Therefore, mRNA vaccine generates protein products with endogenous characteristics, ensuring the precision of targeting.

### Induction of both innate and adaptive immunities

Efficiency is another keypoint in precision therapy. Meeting the requirement, mRNA vaccine can induce both innate and adaptive immunities to exert efficient anti-tumor effects. Innate immunity forms the first line of defense against non-self antigens [97]. APCs, especially DCs, engulf foreign mRNA via pattern recognition receptors (PRRs), activating a series of proinflammation-related signaling pathways that promotes the function of innate immunity [97, 99]. For example, PRR toll-like receptor (TLR)-3 recognizes and binds double-strand RNA, regulating the secretion of cytokines and chemokines as well as the activation of the type I interferon (IFN) pathway [100]. In addition, PRR TLR-7 and TLR-8 bind single-strand RNA, activating nitric oxide synthase and the production of type I IFN [101–103]. The secretion of type I IFN is essential for the formation of an immune-stimulatory environment, wherein T cells differentiate into cytotoxic types that can eliminate tumors. Apart from innate immunity, mRNA vaccines further stimulate adaptive immunity. The protein encoded by non-self mRNA can be degraded into peptides, which are routed into the endoplasmic reticulum, loaded onto MHC-I, shuttled to the cell surface, and ultimately presented to and activate CD8^+^ T cells [98, 104, 105]. Meanwhile, the antigens can be transported from Golgi to endosomes and enter the MHC-II presentation pathway, where they activate CD4^+^ T cells [106]. Actually, the antigens can also be secreted and reinternalized and presented via MHC-II to activate CD4^+^ T cells or cross-presented via MHC-I to activate CD8^+^ T cells [104, 106]. Moreover, mRNA vaccine can upregulate the expression of costimulatory molecules (e.g., CD40 and CD86) on APCs (e.g., DCs), enhancing the antigen presentation and T cell activation [107]. Furthermore, activated APCs (e.g., macrophage and DC) present antigens to activate B cells, triggering an antibody response [108, 109]. Multiple preclinical and clinical trials have shown that mRNA vaccines induce antitumor immune responses and tumor rejection. Melanoma mouse models have revealed that mRNA-lipoplexes encoding mutant or viral neo-antigens or endogenous self-antigens trigger IFN-α release by macrophages and plasmacytoid DCs, induce strong effector and memory T-cell responses, and mediate the rejection of progressive tumors [107]. A personalized mRNA vaccine induces T cell infiltration and specific killing of melanoma [43]. Additionally, the intravenously administered liposomal RNA vaccine BNT111 mediates a durable objective response and induces strong anti-melanoma CD4^+^ and CD8^+^ T cell immunity after pretreatment with an immune checkpoint inhibitor [110]. In summary, mRNA vaccines accord with the concerns about efficiency in precision therapy, inducing both innate immunity and adaptive immunity to exert potent anti-tumor effects.

### High safety in practice

Safety is also important for precision therapy. In line with this, mRNA production does not involve toxic chemicals and the risk of contamination with the adventitious virus packaged in cell cultures. Therefore, an mRNA approach averts common threats associated with other vaccine platforms (e.g., viral vectors, inactivated viruses, live viruses, and subunit protein vaccines). In addition, the rapid manufacturability of mRNA decreases opportunities for the introduction of contaminating microorganisms. In this context, it is also important to note that mRNA cannot integrate into the host genome, ruling out oncogenic potentials. Finally, mRNA can be rapidly degraded by RNA enzymes and is characterized by its adjustable half-life, which defines the controllable expression of mRNA-encoded proteins [111–113]. The first clinical study of mRNA vaccine was conducted in 2008 in melanoma patients [114]. Vaccination with naked mRNA is safe and well tolerated and does not induce World Health Organization grade III or IV adverse events. Numerous clinical trials have supported the high safety of mRNA vaccines [115–117]. For example, direct injection of protamine-protected mRNA into patients with metastatic melanoma predominantly caused local inflammatory skin reactions or fatigue, which could be easily lessened by symptomatic therapy. Therefore, mRNA vaccines are relatively safe, which is consistent with the principle of safety underlying precision therapy.

### Convenience and low cost of preparation

Economic principle is last, but perhaps most important, in precision therapy. Exactly, preparation of mRNA vaccine is convenient and low cost. mRNA can be produced in vitro using a DNA template, ribonucleotide triphosphates, and recombinant enzymes [118, 119]. In this process, a plasmid DNA containing a DNA-dependent RNA polymerase promoter (e.g., T3, T7, or SP6) is first generated. It is then linearized to provide a template for mRNA synthesis using DNA-dependent RNA polymerase before degradation by DNase. A 5′cap and a 3′poly-A tail are added during the transcription step to facilitate efficient translation in vivo. Finally, free nucleotides, enzymes, truncated RNA fragments, and residual DNA are removed to obtain pure mRNA. This simple process ensures rapid mRNA production in a relatively less complex system and thus can be standardized to produce almost any encoded protein immunogen, rendering it highly suitable for constructing personalized vaccines for cancer treatment. Moreover, all reaction components and enzymes required for mRNA production are commercially available. The entire process of mRNA vaccine production takes about ten days, significantly shorter than other formats [53]. The rapid production of mRNA vaccine is a tremendous advantage for personalized therapy, meaning that treatment can be available within a short time after diagnosis. From an industrial perspective, the large-scale production of mRNA vaccines is low-cost. DNA templates are used during the transcription cycle and by scaling the in vitro transcription reaction. A very small (about 1 µg) DNA template can produce very large amounts (hundreds µg) of capped mRNA, and the product is more dependent on the transcription volume and time than on the amount of DNA [120]. In addition, the required mRNA vaccine dose is generally lower than DNA vaccines (50–100 µg for an mRNA vaccine and 1–5 mg for a DNA vaccine). Actually, an only 10 g of mRNA can generate about 100,000 vaccine doses. Together, the production of mRNA vaccines is convenient and low-cost, which is in agreement with the economic principle of precision therapy.

## Development strategy of personalized mRNA vaccines for pancreatic cancer

**Fig. 2:**
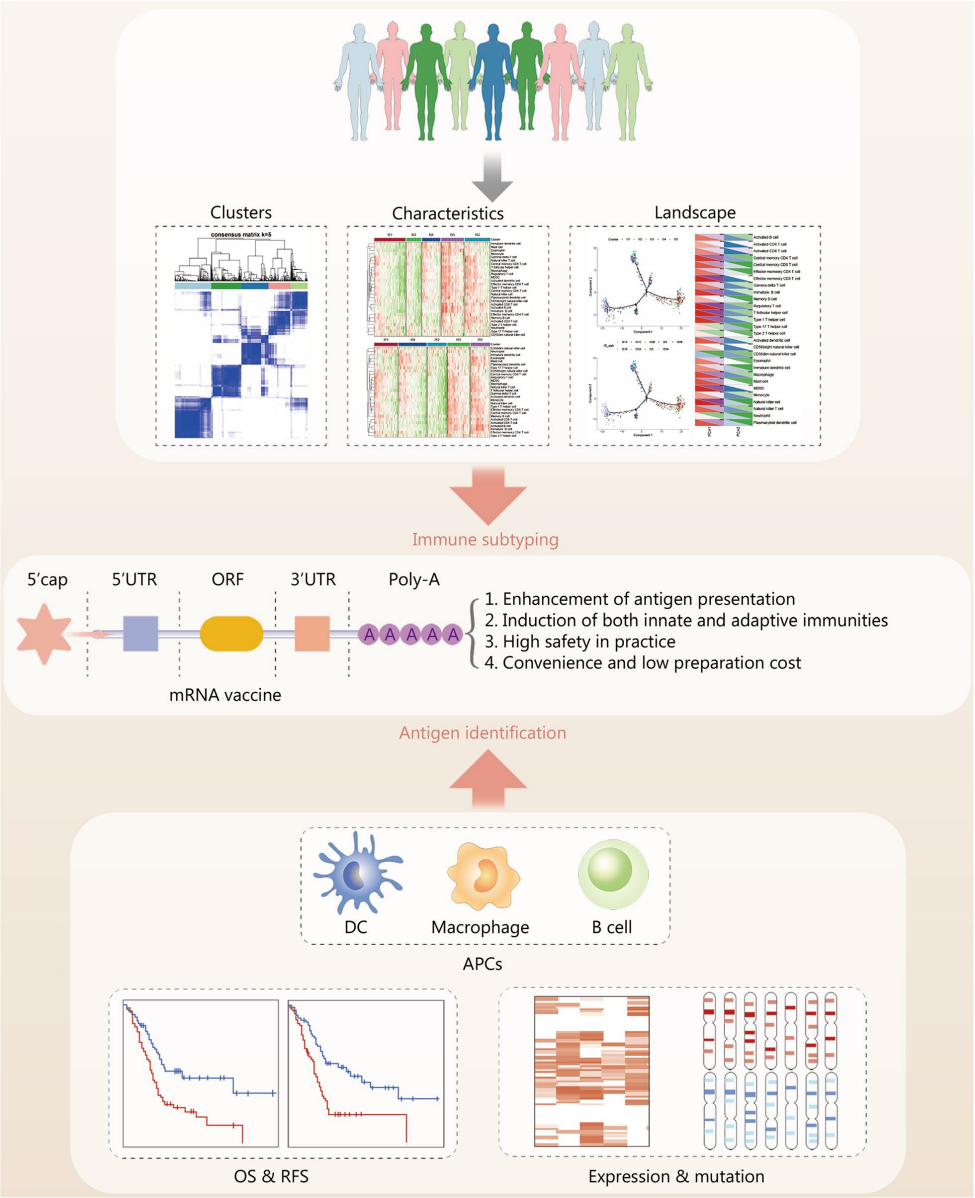
Streamlined development of personalized mRNA vaccines for pancreatic cancer. Novel tumor antigens are identified as potent targets for the preparation of promising pancreatic cancer mRNA vaccines. Immune subtypes are identified as vital criteria for selecting applicable pancreatic cancer patients for mRNA vaccine treatment. Partial elements of this figure are adopted from Huang et al. [29] with appropriate modification. ORF open reading frame, DC dendritic cell, APCs antigen-presenting cells, OS overall survival, RFS relapse-free survival

With precise targeting, efficiency, safety, and economic cost, mRNA vaccines offer promise for providing personalized pancreatic cancer treatment. Accumulating evidence suggests that the pipeline for developing personalized pancreatic cancer mRNA vaccines should be divided into three critical modules, including identifying tumor antigens, constructing mRNA vaccines, and distinguishing immune subtypes (Fig. 2).

### Identification of pancreatic cancer antigens

Antigen selection is the first step in developing a vaccine. An ideal vaccine candidate should possess the following characteristics: unique to tumor cells, involved in tumorigenesis and progression, non-tolerated by the immune system, and stimulatory to the antitumor immunity [121–123]. Current immunogenic targets for cancer treatment include tumor-associated antigens (TAAs) and tumor-specific antigens (TSAs) [124]. TAAs are the most commonly targeted antigens typically expressed on normal cells but aberrantly on tumor cells. This underscores their potential as universal therapeutic targets, although they are self-antigens and thus may be immunologically tolerated and weakened vaccine potency. Unlike TAAs, TSAs are exclusively expressed in tumor cells with strong immunogenicity and a high degree of individuality and epitope diversity and thus are ideal targets for personalized vaccines. Engineering a personalized anti-pancreatic cancer mRNA vaccine begins with identifying tumor-specific non-synonymous mutations by comparing next-generation sequencing data of tumors and paired normal tissues [125]. Computational neoantigen prediction pipelines are then applied to verify the expression and predict the binding affinity of peptides generated from mutated genes onto MHC alleles. High transcript expression is related to enhanced T cell response and can compensate for the low MHC-binding affinity of mutations [126]. Furthermore, a single MHC-I-bound TSA is not sufficient, and additional MHC-II-bound TSAs are needed for effective antitumor immunity [127]. NetMHCpan and MHCflurry are tools trained for predicting the binding affinity between ligands and MHC [128–130]. Notably, for predicting immunogenicity, the stability of the neoepitope-MHC complex is more important than the binding affinity [131]. NetMHCstabpan, a tool for stability prediction, performs well in identifying immunogenic mutations [132]. In addition to the surface presentation, the interaction between peptide-MHC complex and T-cell receptor is necessary to induce an immune response, and predicting this interaction is based on amino acid side chains of the T-cell receptor facing the MHC-bound peptide [133]. Recently, a perspective pipeline for identifying tumor antigens by screening for the overexpressed and mutated genes and prognosis and APC-associated candidates has been established [29]. Notably, although the above-mentioned characteristics are based upon a sound rationale, a specific approach to weigh each of them has not been set up, and therefore, optimal candidates for mRNA vaccine development cannot be selected. Nevertheless, tumor antigen prediction is rapidly evolving thanks to the recent progress in computational biology. Accordingly, an accurate and sensitive approach for identifying potent candidates in individual pancreatic cancer will eventually be established for developing its personalized mRNA vaccines.

### Construction of mRNA vaccines against pancreatic cancer

Several critical issues, including delivery, stability, translation, and immunogenicity, must be addressed before the practical application of the mRNA-based cancer vaccine [97, 98, 134, 135]. Because of its size, degradability, and charge, naked mRNA cannot efficiently pass through the cell membrane and enter the cytoplasm, except for immature DCs that can efficiently uptake mRNA via the macro-pinocytosis pathway [134, 136]. For more effective delivery of mRNA into APCs, mRNA formulations (e.g., liposomes, polyplexes, polysomes, and lipoplexes) and administration routes must be appropriately selected and optimized. After successful mRNA delivery, the half-life of mRNA transcribed in vivo must be appropriately regulated, given that several factors influence the pharmacodynamic and pharmacokinetic properties of mRNA-based therapeutics. There is a need to improve mRNA structures, including optimization of poly (A), 5′cap, poly-A tail, untranslated regions, and protein-encoding open reading frames, to enhance the stability of mRNAs [115, 137, 138]. In addition to delivery and stability, immunogenicity must also be considered. Accumulating evidence suggests that there is a negative feedback loop between mRNA and it-induced immune response. For instance, exogenous RNA stimulates the production of type I IFN by stimulating innate immunity [98], while excessive production of type I IFN inhibits translation and promotes the degradation of both ribosomal RNA and cellular mRNA [98, 134, 139, 140]. The addition of poly-A tails, optimization of sequences, and posttranscriptional purification can decrease the level of innate immunity without altering the translation of mRNA [112, 141–145]. Furthermore, increasing the immunostimulatory properties of mRNA using adjuvants promotes the potency of cancer mRNA vaccines. TriMix (mRNA encoding CD70, CD40L, and TLR4) enhances the immunogenicity of unmodified naked mRNA and improves the cytotoxicity of T lymphocyte and DC maturation [98]. Together, advances in optimization strategies for mRNA vaccine construction largely improve the efficacy of these vaccines for pancreatic cancer treatment.

### Distinction of immune subtypes in pancreatic cancer

**Fig. 3:**
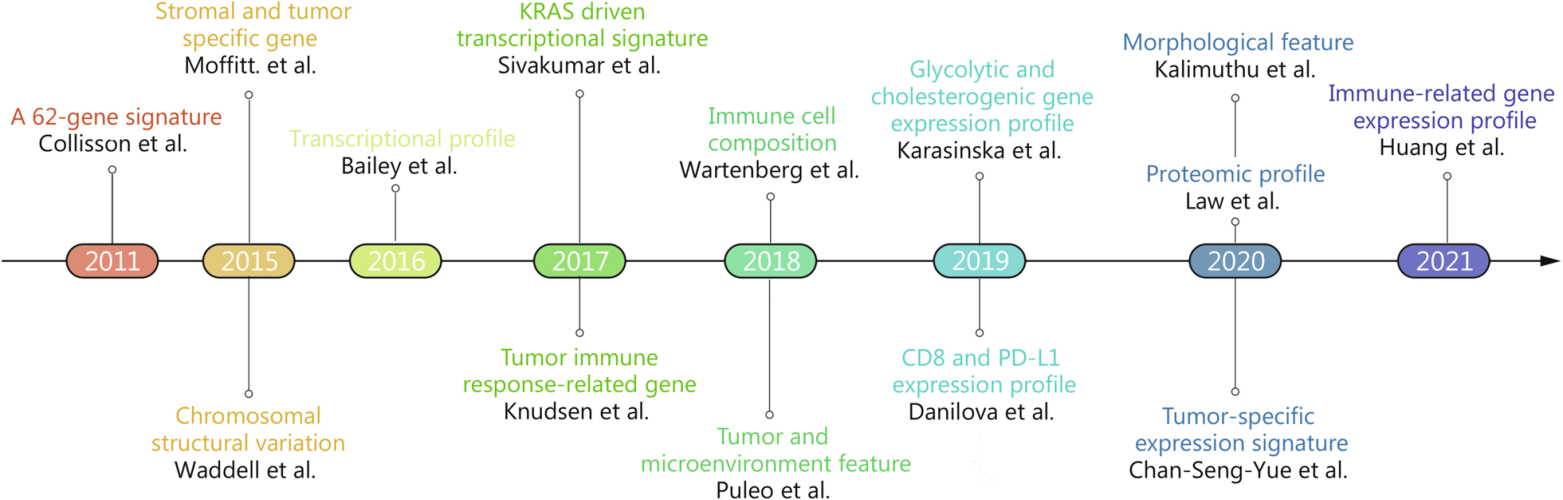
Timeline of pancreatic cancer subtyping. The timeline of pancreatic cancer subtyping, together with the distinct classification approaches and the corresponding authors, including Collisson et al. [151], Moffitt et al. [28], Waddell et al. [152], Bailey et al. [153], Sivakumar et al. [154], Knudsen et al. [155], Wartenberg et al. [150], Puleo et al. [156], Karasinska et al. [157], Danilova et al. [149], Kalimuthu et al. [158], Law et al. [159], Chan-Seng-Yue et al. [31], and Huang et al. [29], are shown as indicated. KRAS GTPase KRas, PD-L1 programmed cell death ligand 1

Pancreatic cancer is usually characterized by a complex immunosuppressive microenvironment, low mutational burden, and poor T cell infiltration [11, 146, 147]. Although an mRNA vaccine can activate and promote infiltration of T cells into the tumor, the entry of these cells could still be largely interfered with by the desmoplastic stroma of pancreatic cancer cells [4, 148]. Moreover, numerous immunosuppressive cells (e.g., myeloid cells, regulatory T cells, and M2 macrophages), signaling pathways (e.g., transforming growth factor beta signaling pathway, IL-10 signaling pathway, and VEGF signaling pathway), and molecules (e.g., PD-L1, T cell immunoglobulin mucin 3, T cell immunoreceptor with Ig and ITIM domains, lymphocyte activating 3, V-type immunoglobulin domain-containing suppressor of T-cell activation, and CD73) lead to multiple immunosuppression on anti-tumor immune response in the pancreatic cancer microenvironment [11, 146]. Hence, mRNA vaccines in combination with other therapies (rather than a single vaccine) and biomarkers for predicting therapeutic response of combination strategies are strongly needed for pancreatic cancer treatment. To date, diverse pancreatic cancer subtypes, defined based on different parameters, approaches, and perspectives, have been identified (Fig. 3). Multiple immunological factors, including immune-related gene expression profile and immune cell composition, are used for grouping immune subtypes of pancreatic cancer. Immune subtypes indicate the immunological status in pancreatic tumors and their microenvironment and thus are accurate biomarkers for selecting a suitable combined therapy [29, 149, 150]. For instance, immunologically "cold" pancreatic tumors generally show low immunogenicity and/or high reactive stroma, whereas immunogenic chemotherapy and stromal modulation may promote the effectiveness of an mRNA vaccine by improving tumor immunogenicity and T cell infiltration. Additionally, an mRNA vaccine combined with immune checkpoint blockade may improve T cell infiltration and function in immunologically "hot" tumors. Overall, combination therapy may enhance the efficacy of an mRNA vaccine for pancreatic cancer treatment under the guidance of immune subtypes, biomarkers for matching patients and therapeutics.

## Concluding remarks and outlook

The mRNA vaccine is a novel and promising vehicle for developing personalized vaccines against pancreatic cancer. Identification of potent tumor antigens, optimization of immunostimulatory vaccine construction, and distinction of immune subtypes are prerequisites for the personalization of potentially effective pancreatic cancer mRNA vaccines.

At present, a first-in-human phase I study on the tolerability and safety of the mRNA-based personalized neoantigen vaccine (autogene cevumeran, also known as BNT122, RO7198457) in combination with chemotherapy and PD-L1 blockade for resected PDAC is currently underway. The preliminary findings in this trial were released for the first time on June 5, 2022 by BioNTech Company (https://investors.biontech.de/). Sixteen patients who underwent surgery and received PD-L1 inhibition were vaccinated with autogene cevumeran, and all well tolerated the treatment. Only one developed a vaccine-related grade three fever and hypertension, and no other grade three or higher adverse events were observed. In addition, half were detected de-novo neoantigen-specific T cell responses, and had a significantly longer recurrence-free survival (median not determined, but is more than 18 months) compared with those without vaccine-induced immune responses (13.4 months). Therefore, personalized mRNA vaccine is a promising strategy for pancreatic cancer treatment. Notably, only about 15–20% of pancreatic cancer patients present with localized disease that can be resected through the standard procedure [3]. In contrast, the majority of pancreatic cancers are diagnosed at the locally advanced or metastatic level and/or are poorly differentiated and ineligible for surgery, rendering the acquirement of these tumors largely dependent on biopsy. However, the inter- and intra-tumoral heterogeneity of pancreatic cancer limits the actual value of biopsy-derived samples, at least not sufficient for the personalized design and construction of mRNA vaccines as well as the distinction of patients for suitable combination therapy. Neoadjuvant therapies can be used to reduce tumor staging and eliminate micro-metastases, increasing the chance of successful surgery [160–162]. For instance, after treatment with the neoadjuvant FOLFIRINOX, 76 of 125 (60.8%) patients with unresectable pancreatic cancer qualified for tumor resection [160]. In a separate study, 141 patients with unresectable (51.1%) or borderline-resectable (48.9%) non-metastatic cancers were recruited; of these, 78% qualified and underwent surgery after FOLFIRINOX therapy [161]. Therefore, neoadjuvant therapies may facilitate the acquisition of relatively sufficient tumor samples for identifying individualized tumor antigens and immune subtypes for developing personalized mRNA vaccines.

**Fig. 4:**
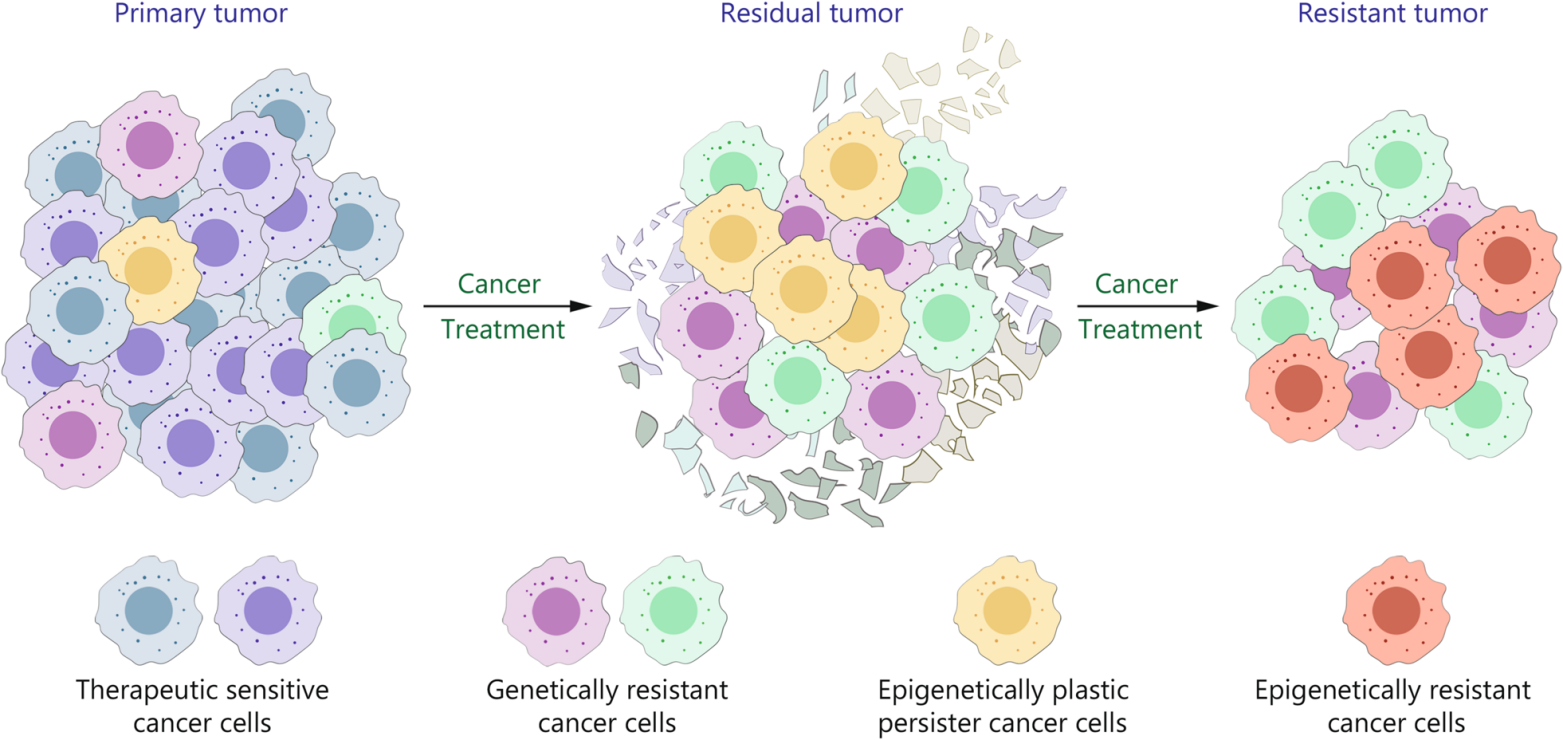
Tumor evolution in pancreatic cancer therapy. Pancreatic cancer is characterized by prevalent intra-tumoral heterogeneity and composed of therapeutic sensitive cells, genetically resistant cells, and epigenetically persister cells. Therapeutic stress in pancreatic cancer causes the transformation of tumor characteristics, leading to acquired resistance

Nevertheless, treating pancreatic cancer with mRNA vaccines remains challenging. As mentioned above, pancreatic cancer is highly heterogeneous. Due to the complexity of the pancreatic tumor components, including both therapeutically sensitive cancer cells and genetically resistant cancer cells or epigenetically plastic persister cancer cells, the therapeutic stress may cause tumor evolution and consequent treatment failure (Fig. 4). Alternatively, the development of prophylactic mRNA vaccines may be another considerable strategy against this intractable disease. To date, prophylactic mRNA vaccination is mainly applied for preventing infection of viruses, since they are ectogenic and possess simple construction and antigens for vaccine development can easily be identified. In contrast, the development of prophylactic vaccines against pancreatic cancer is still in infancy, partially but not totally, due to the complexity of pancreatic cancer onset and the difficulty of assessing the effectiveness of vaccination. Research shows that PDAC arises from non-invasive precancerous lesions, microscopic pancreatic intraepithelial neoplasia (PanIN), and macroscopic intraductal papillary mucinous neoplasms (IPMNs) [163]. In processing PanIN-PDAC transformation, oncogenic mutation in *KRAS* gene has been detected in > 90% of the low-grade-PanINs [163]. In IPMN-PDAC progression, oncogenic mutations in *KRAS* and *GNAS* genes have been observed in 50–80% and 40–70% of IPMN, respectively [164]. KRAS and GNAS are therefore considered potential targets for preventing PDAC development. A KRAS-targeting peptide vaccine for preventing pancreatic cancer in high-risk individuals is currently under clinical test (NCT05013216), but its prophylactic efficiency is still unknown. Notably, apart from *KRAS* mutation, multiple genetic and epigenomic mechanisms jointly contribute to pancreatic cancer initiation. For instance, IL-33 is identified as a key factor that induces epigenetic reprogramming-mediated pancreatic oncogenesis [165, 166]. The question that whether co-targeting KRAS and IL-33 increases the probability of pancreatic cancer prevention arises and remains to be validated.

## Data Availability

Data sharing is not applicable to this article as no datasets were generated or analyzed during the current study.

## Abbreviations

APC: Antigen-presenting cell
COVID-19: Coronavirus disease 2019
DC: Dendritic cell
ENO1: Enolase 1
GM-CSF: Granulocyte–macrophage colony-stimulating factor
HSPPC-96: Heat shock protein-peptide complex-96
IFN: Type I interferon
IPMNs: Intraductal papillary mucinous neoplasms
MHC: Major histocompatibility complex
mRNA: Messager RNA
MUC1: Mucin 1
PanIN: Pancreatic intraepithelial neoplasia
PDAC: Pancreatic ductal adenocarcinoma
PD-1: Programmed cell death 1
PD-L1: Programmed cell death ligand 1
PPRs: Pattern recognition receptors
SARS-CoV-2: Severe acute respiratory syndrome coronavirus-2
TAAs: Tumor-associated antigens
TEXs: Tumor-derived exosomes
TLR: Toll like receptor
TSAs: Tumor-specific antigens
VEGFR: Vascular endothelial growth factor receptor
WT1: Wilms tumor 1
